# A behavioural change package to prevent hand dermatitis in nurses working in the national health service (the SCIN trial): study protocol for a cluster randomised controlled trial

**DOI:** 10.1186/s13063-016-1255-y

**Published:** 2016-03-17

**Authors:** Ira Madan, Vaughan Parsons, Barry Cookson, John English, Tina Lavender, Paul McCrone, Caroline Murphy, Georgia Ntani, Lesley Rushton, Julia Smedley, Hywel Williams, Alison Wright, David Coggon

**Affiliations:** Guy’s and St Thomas’ NHS Foundation Trust, Occupational Health Service, St Thomas’ Hospital, Westminster Bridge Rd, London, SE1 7EH UK; King’s College London, London, UK; Royal Free Hampstead NHS Trust, London, UK; Nottingham University Hospitals NHS Trust, Nottingham, UK; University of Manchester, Manchester, UK; University of Southampton, Southampton, UK; Imperial College London, London, UK; University Hospital Southampton NHS Foundation Trust, Southampton, UK; University of Nottingham, Nottingham, UK

**Keywords:** Dermatitis, Occupational health, Intervention, Nurses, Prevention, Randomised controlled trial, Behavioural change, Moisturiser

## Abstract

**Background:**

Hand dermatitis can be a serious health problem in healthcare workers. While a range of skin care strategies and policy directives have been developed in recent years to minimise the risk, their effectiveness and cost-effectiveness remain unclear. Evidence now suggests that psychological theory can facilitate behaviour change with respect to improved hand care practices. Therefore, we will test the hypothesis that a behavioural change intervention to improve hand care, based on the Theory of Planned Behaviour and implementation intentions, coupled with provision of hand moisturisers, can produce a clinically useful reduction in the occurrence of hand dermatitis, when compared to standard care, among nurses working in the UK National Health Service (NHS) who are particularly at risk. Secondary aims will be to assess impacts on participants’ beliefs and behaviour regarding hand care. In addition, we will assess the cost-effectiveness of the intervention in comparison with normal care.

**Methods/Design:**

We will conduct a cluster randomised controlled trial at 35 NHS hospital trusts/health boards/universities, focussing on student nurses with a previous history of atopic disease or hand eczema and on nurses in intensive care units.

Nurses at ‘intervention-light’ sites will be managed according to what would currently be regarded as best practice, with provision of an advice leaflet about optimal hand care to prevent hand dermatitis and encouragement to contact their occupational health (OH) department early if hand dermatitis occurs. Nurses at ‘intervention-plus’ sites will additionally receive a behavioural change programme (BCP) with on-going active reinforcement of its messages, and enhanced provision of moisturising cream.

The impact of the interventions will be compared using information collected by questionnaires and through standardised photographs of the hands and wrists, collected at baseline and after 12 months follow-up. In addition, we will assemble relevant economic data for an analysis of costs and benefits, and collect information from various sources to evaluate processes.

Statistical analysis will be by multi-level regression modelling to allow for clustering by site, and will compare the prevalence of outcome measures at follow-up after adjustment for values at baseline. The principal outcome measure will be the prevalence of visible hand dermatitis as assessed by the study dermatologists. In addition, several secondary outcome measures will be assessed.

**Discussion:**

This trial will assess the clinical and cost effectiveness of an intervention to prevent hand dermatitis in nurses in the United Kigdom.

**Trial registration:**

ISRCTN53303171: date of registration, 21 June 2013.

## Background

Occupational irritant hand dermatitis is a major risk in healthcare. In a recent study, the 1-year prevalence of self-reported hand dermatitis among healthcare workers in a Dutch university medical hospital was 24 %, as compared with less than 10 % in the general population [1]. Amongst healthcare workers, nurses are the group at highest risk of hand dermatitis, with an estimated point prevalence of 18–30 % [2, 3]. Moreover, in a study of German geriatric nurses, two thirds of those who reported hand dermatitis stated that it had developed after they had joined the profession [2]. Consistent with this, among Korean nursing students, the prevalence of hand dermatitis increased from 7 % in the first year to 23 % in the fourth year of training [4]. The costs of hand dermatitis to the individual and employer are high. It not only affects quality of life, but also can lead to loss of employment [5, 6]. Once an individual has developed irritant hand dermatitis the prognosis is poor. In a 15-year follow-up study of a Swedish general population sample, about a third of those with hand dermatitis needed on-going medical treatment and 5 % experienced long periods of sickness absence, loss or change of job, or ill-health retirement [7]. Affected individuals may also experience negative psychosocial consequences, such as sleep disturbance and interference with leisure activities [7].

The high prevalence of hand dermatitis in nurses is attributed to frequent hand-washing and poor hand-drying techniques [8]. Current hand-cleansing policies in the UK National Health Service (NHS) are driven by efforts to reduce bacterial colonisation and transmission of infections, and the emphasis is on frequent use of hand rubs before and after patient contact, and washing with soap and water if the hands are visibly soiled [8]. However, little attention is paid to prevention of hand dermatitis.

For a nurse who develops irritant hand dermatitis, the condition is likely to be aggravated by exposure to hand hygiene measures. The presence of hand dermatitis may discourage nurses from undertaking adequate hand decontamination due to discomfort or concern about exacerbating skin lesions. It is known that 50 % of people with hand dermatitis are colonised with *Staphylococcus aureus* [9], and although controversial, there is a theoretical risk that nurses with hand dermatitis infected by methicillin-resistant *Staphylococcus aureus* (MRSA) could transmit the infection to patients. Occupational health (OH) professionals often have to advise nurses with active dermatitis to refrain from work until the lesions are healed, as it is difficult for them to avoid frequent hand-washing unless they are redeployed to a non-clinical area.

Various measures might help to prevent hand dermatitis in nurses and reduce the problems that it causes.

### Moisturisers

Two systematic reviews of the management of occupational dermatitis [10, 11] have concluded that moisturisers contributed importantly to both prevention and treatment at work. A review by the former Occupational Health Clinical Effectiveness Unit at the Royal College of Physicians focussed on the evidence for managing established occupational dermatitis, as distinct from prevention [10]. It found inconsistent evidence from two studies in which moisturisers had been used as part of a complex intervention in nurses [12, 13], but concluded that there was sufficient evidence to recommend that skin care programmes should include the use of emollients.

Guidelines produced by the British Occupational Health Research Foundation [11] recommended that the regular application of emollients helps to prevent the development of occupational dermatitis, citing three high-quality studies [14–16]. One randomised control trial (RCT) found an improvement in all outcomes, including clinical skin inspection. In the other, transepidermal water loss (TEWL) improved among construction workers who used pre- and after-work creams compared to controls, but there was no difference in clinically assessed skin condition [15]. Moisturisers also improved skin condition in workers with damaged skin [17]. More recent reviews have also concluded that there is some evidence to support the use of educational interventions that include moisturisers, but this came from a small number of workplace studies, and the authors strongly recommended that further large high-quality RCTs in working groups were needed [18, 19].

In the experience of the dermatologists and OH physicians in our research team, moisturisers are not widely used by healthcare workers in the UK. This anecdotal observation is supported by a study of nurses working in intensive care units (ICUs) in Germany which found that only 15 % of the 204 respondents reported that they applied moisturising creams after hand- washing and only 2 % after skin disinfection with hand rubs. Furthermore, 9 % never applied skin care to their hands and 72 % reported that they did not perform final skin care after the last hand-wash of the day [20].

### Hand cleansing

The use of antibacterial hand rub with the addition of moisturisers for hand hygiene reduces the drying and cracking of the skin that commonly results from repeated hand cleansing with soap and water [21]. In addition, antibacterial hand rubs are associated with increased hand hygiene compliance and reduced rates of nosocomial infection [22].

### Hand-drying and glove use

Proper drying of the hands after washing is pivotal to good hand hygiene and care, particularly as wet skin is more likely to facilitate the transmission of bacteria than dry skin. A recent review of hand-drying processes [23], which included 12 studies, concluded that paper towels are superior to electric air dryers and, therefore, should be recommended in locations where hygiene is vital, such as clinical environments. This was supported by guidelines from the World Health Organization [8].

Skin care programmes that incorporate measures of the type that have been described have shown a beneficial effect in the prevention of hand dermatitis in healthcare workers [12, 13, 24, 25]. However, a recent systematic review suggested that educational programmes could benefit from being more strongly informed by psychological theory, since their success relies on employees adopting appropriate preventive and protective behaviours [18]. Psychological theory has proved useful in understanding the behavioural determinants of hand hygiene practices among healthcare professionals [8, 26] and so is likely to also be useful in the design of interventions to modify such practices. Moreover, a meta-analysis of Internet-based behavioural change interventions found that more extensive use of theory was associated with significantly greater effects and, in particular, that Internet interventions based on the Theory of Planned Behaviour (TPB) tended to have more substantial effects on behaviour [27]. One of the few studies applying psychological theory to the prevention of occupational hand dermatitis examined the TPB’s ability to predict the behaviour of a sample of German patients with occupational hand dermatitis receiving an inpatient tertiary prevention programme. The TPB variables explained 30 % of the variance in post-intervention dermatitis prevention behaviour and 38 % of the variance in intentions for preventive behaviours [28]. Systematic review of relevant evidence shows that forming implementation intentions and specific plans about how, when and where health-promoting behaviours will be performed increases the likelihood of individuals acting on their positive intentions [29]. Furthermore, evidence suggests that reminding individuals of their implementation intentions can facilitate longer-term changes in behaviour [30, 31].

Although there are good reasons to expect that well-designed skin care programmes would be beneficial for nurses, their effectiveness and cost-effectiveness remain uncertain. Trials to date have been limited by size and the possibility that the control group was aware of the intervention [32], or by a failure to address cost-effectiveness [33]. There is a need for a pragmatic trial to evaluate the clinical and cost-effectiveness of a behavioural change programme (BCP) to improve the compliance of nurses with measures to prevent occupational hand dermatitis.

## Aims and objectives

1. We will test the hypothesis that a bespoke, web-based behavioural change intervention to improve hand care, coupled with provision of hand moisturisers, can produce a clinically useful reduction in the prevalence of objectively assessed hand dermatitis after 1 year, when compared to standard care, among nurses working in the National Health Service (NHS) who are particularly at risk
2. Secondary aims will be to assess impacts on participants’ beliefs and behaviour regarding hand care (as a measure of adherence), days off sick over a 1-year follow-up period, and use of hand moisturisers
3. In addition, we will assess the cost-effectiveness of the intervention compared with normal care

## Method/Design

### Concise statement of proposed research

We will conduct a cluster RCT of an intervention to improve hand care at 35 sites (12 NHS acute hospital trusts/health boards which provide OH care to both student and ICU nurses, 18 NHS trusts which provide OH care to ICU nurses and 5 university OH departments which provide OH care to student nurses). We will focus on two groups of staff: (1) student nurses who are about to start their first clinical placements, and who are at increased risk of hand dermatitis because of a past history of atopic disease or hand eczema; and (2) nurses working in ICUs, who are at increased risk of hand dermatitis because of the nature of their work.

Ethics approval to conduct the trial has been granted by the National Research Ethics Service (NRES) Committee London − City Road and Hampstead (REC reference: 13/LO/0981). Ethical approval applies to all research sites taking part in the study. Trial registration: ISRCTN number 53303171: date of registration, 21 June 2013.

### Design and theoretical/conceptual framework

The study will be a cluster RCT with sites as the unit of randomisation. Sites will be randomly allocated to be ‘intervention-light’ or ‘intervention-plus’. Study group 1 will be student nurses who are about to start their first clinical placements, and who are at increased risk of hand dermatitis because of a past history of atopic disease or hand eczema. Study group 2 will be nurses working in ICUs who are at increased risk of hand dermatitis because of the nature of their work. All participants are required to provide written consent at the time of recruitment into the study.

### Intervention-plus

The intervention-plus in both staff groups will centre on a bespoke online behavioural change package (BCP) which will include advice: on when and when not to use gloves; on when to use antibacterial hand rubs; on when to use moisturising cream; and to contact OH early if hand dermatitis occurs.

The BCP has been developed by members of the study team with expertise in dermatology, occupational medicine, nursing, and health psychology. Care has been taken to ensure compatibility with current guidance on infection control. We have emphasised WHO recommendations that hands should only be washed with soap and water if visibly soiled. At all other times, hands should be cleansed with antibacterial hand rubs [8].

To maximise the probability of participants following best practice, they will be asked to form implementation intentions for performing each of the behaviours in their workplace. A record of each participant’s implementation intentions will be generated by the online BCP programme and emailed to them. In the event of a participant being unable to access the online BCP, they will be posted a paper-based magazine version of the BCP which will reflect the information provided in the online BCP. Participants will asked to read through the material provided and write down their action plans in spaces provided. They will then be asked to keep the paper-based BCP in a convenient place so that they can refer back to it as required.

The BCP will be supported by provision of facilities to encourage adherence. These will include personal supplies of moisturising cream for at-risk student nurses, and provision of (1) optimal equipment for cleaning hands, and (2) moisturising cream dispensers on ICUs.

The BCP will be made available to ICU nurses once they have undergone baseline assessment (see below), and to student nurses 2 weeks before they start their first clinical attachment. It will be actively reinforced over the course of the study by consistent messages on skin care from the local OH and control of infection teams, and from local line management. Research has shown that senior role models have important effects on more junior healthcare workers’ hand hygiene behaviours [34, 35], and it seems reasonable to assume that this influence will extend to behaviours that prevent dermatitis. To facilitate this, when implementing intervention-plus, we will engage in a dialogue with local OH staff and line managers about the nature and purpose of the study and will provide them with information on the advice which will be given in the BCP, to ensure that they promote consistent messages on skin care. The SCIN research team will also write to any NHS sites at which student nurses undertake clinical placements, but which are not themselves participating study sites, to ensure they are informed about the study.

We will offer the BCP online to allow nurses to access it at a time convenient to their schedules, to permit standardisation of the delivery of key information across all intervention- plus sites, and to reduce the potential burden on OH staff. Moreover, if the BCP is found to be effective in this trial, it will be simple to scale up access to the website in order to deliver the BCP across the country.

### Comparator (intervention-light)

Nurses at intervention-light sites will be managed according to what would currently be regarded as best practice, with provision of an advice leaflet about optimal hand care entitled ‘Dermatitis: occupational aspects of management. Evidence-based guidance for employees’ (also provided to the intervention-plus group) and encouragement to contact their OH department early if hand dermatitis occurs. However, they will not receive the BCP or active reinforcement of its messages. Nor will they routinely be offered supplies of moisturising cream over and above what is already standard practice at their site.

### Sampling

We identified all NHS sites in the UK that train nurses, have an in-house OH service, and have at least one ICU. We first wrote to the lead occupational physician in each eligible site in December 2011, asking their willingness, in principle, to collaborate in a trial; and we wrote again in May 2012 and January 2014 asking them to confirm their willingness to collaborate. We also invited additional sites to sign up to the study via national OH newsletters. This included some sites that had an ICU but did not train nurses, and vice versa. In order to avoid the risk of student nurses moving placements from an intervention to a control site (or vice versa) during the study period, we generally invited only one site in each city or town to participate in the study. The exceptions were London and Manchester. In London, three sites were identified in which student nurses did not move to neighbouring sites during their training. In Manchester, we identified sites where students at three local universities undertake their clinical placements during their first year of nursing training, and ensured that these sites were clustered appropriately to prevent cross-contamination in the study.

A summary of the number of participating sites is provided below:

- 12 sites recruiting both ICU nurses and student nurses (NHS trusts/health boards)
- 18 sites recruiting ICU nurses only (NHS trusts/health boards)
- 5 sites recruiting student nurses only (university-based OH departments)

#### Study group 1 – student nurses

All student nurses are required to attend for OH assessment before starting their clinical work. At participating sites, with permission from the universities concerned, all student nurses in one or more year groups (excluding mental health nursing students) who are due to start their first clinical placement, will be sent a Participant Information Sheet by their university or their OH department before or at the time of their mandatory assessment. Those who have a history of atopic disease or hand eczema will be identified by the OH department at the assessment, from information that they provide in a generic pre-placement health screening questionnaire. An OH clinician will explain to them that because of their constitution, they are at increased risk of hand dermatitis and, therefore, need to take special care of their hands. Those student nurses who meet the inclusion criteria will then be invited to participate in the study. A field worker will obtain written consent from those nurses who agree (the lead OH practitioner at the study site and the trial manager being available if needed to answer any questions). The consent form will ask participants to provide a preferred email address and telephone number to facilitate follow-up, and so that those at intervention-plus sites can be sent a link to the online BCP. One copy of the signed consent form will be filed in the nurse’s OH notes, one copy will be sent to the trial manager, and a third copy will be given to the participant. Participants will also be provided with an information sheet to give to their general practitioner.

#### Study group 2 – intensive care nurses

The investigators, trial manager and lead OH clinicians from each site will identify all ICUs (and a few also identified their special care baby units) that would be suitable for the study. A local OH clinician or senior ICU nurse will explain to all nurses working on the selected ICUs that they are at increased risk of hand dermatitis because of frequent hand-washing with cleansers and water. They will also be told that a study is being carried out to help optimise management of nurses who are higher risk. Full-time ICU nurses in the ICU (those working at least 30 hours per week) will be provided with a Participant Information Sheet and will be given up to a week to decide whether they wish to participate in the study. Consent to take part will be obtained by the field worker, who along with the trial manager, will be available to answer any queries about the study. The consent form will ask participants to provide a preferred email address to facilitate follow-up, and so that those at intervention-plus sites can be sent a link to the online BCP. Participants will be provided with an information sheet to give to their general practitioner.

The study will be presented to both study groups as research to assess the causes, consequences and ways of preventing hand dermatitis in nurses who are at increased risk, either because of a personal history of atopy or eczema, or because of the type of work that they do. However, to minimise the chance of bias, they will not be told that they are in an intervention-plus or intervention-light group. During the recruitment phase, field workers will reinforce the importance that participants be able to commit fully to the study, particularly with respect to the completion of questionnaires (see below).

To check that rates of participation do not differ importantly between sites randomised to the two arms of the study, the recruitment process will be carefully documented. The total numbers of eligible student and ICU nurses will be recorded, as will the numbers who consent to take part. We will also record the numbers who drop out of the study, with the dates of drop out.

### Data handling

Data from the trial will be handled by InferMed MACRO v4 and data entry and handling will be audited.

### Randomisation

Randomisation will be carried out using fixed blocks of two, stratified by small or large site with or without student nurses. Two sites will be randomised at a time and the King’s Clinical Trials Unit will control the order of the randomisation in order to ensure allocation concealment. Baseline will be collected prior to randomisation.

For the purpose of analyses by intention-to-treat, the date of entry into the study for each participant will be date when they sign the consent form. Although student nurses can only contribute useful information if they progress to clinical work, in practice very few fail to do so once they have begun their nursing studies.

Members of the study team will be notified of the outcome of randomisation in a blinded or unblinded manner, according to their role in the trial. The trial statistician (GN), methodologist (DC), and the dermatologists (HW, JE) will remain blinded to treatment allocation until after the primary analysis.

### Sample size

Field workers will be encouraged to recruit as many eligible student nurses and ICU nurses as possible, with the aim of recruiting at least 40 student nurses and 40 nurses from the ICUs at each site.

To give an indication of power, we assumed that at the end of follow-up the expected prevalence rates overall at the intervention-light sites would be 24 % in both student and ICU nurses, that the intraclass correlation coefficient (ICC) is 0.05 (this corresponds to a variance inflation factor of 2.05 for student nurses and 2.8 for ICU nurses), and that 20 % of participants will be lost to follow-up. With a 5 % level of statistical significance (two-sided), we would have approximately 83 % power to detect a reduction in prevalence of dermatitis at follow-up in the intervention-plus sites to 10 % in student nurses and 91 % power to detect a reduction in prevalence to 10 % at follow-up in ICU nurses. For final prevalence rates of 12 %, the powers would be 68 % for student nurses and 78 % for ICU nurses, while for final prevalence rates of 14 %, the corresponding powers would be 51 % and 61 % respectively. The power will be higher if the ICC is lower than 0.05. (These calculations were carried out using the clustersampsi command in Stata v12.1 for difference in proportions.)

### Setting

OH departments (NHS and university-based) in the United Kingdom (excluding a pilot site in Wales).

### Data collection

#### Study group 1 – student nurses

Student nurses who agree to take part will be asked to complete a self-administered baseline questionnaire (Questionnaire A) covering: contact details (which for security reasons will be kept separate from the rest of the questionnaire); demographic information; history of atopic disease and allergies; activities outside work which predispose to hand dermatitis; beliefs and plans regarding dermatitis prevention behaviours; the EuroQol-5D (EQ-5D) health-related quality of life questionnaire [36] and history of hand dermatitis ever, in the past 12 months, and currently. They will be asked to place the completed questionnaire into a sealed business reply envelope and return it to the SCIN research team in London via the field worker, or if they prefer, directly. In addition, they will be invited to have their hands and wrists photographed.

Participants will be told that they may be asked to access an online BCP, which they should undertake in the week before starting their first clinical attachment, and to which they will be sent a link (by email) 2 weeks before the attachment begins. At that time, they will also be sent (by post) a personal tube of moisturising cream with guidance on how to request further supplies if needed. All participants, at both intervention-plus and intervention-light sites, will be encouraged (orally, through a written advice leaflet, and by email reminders at 4 and 8 months) to attend their OH department at an early stage should they develop hand dermatitis.

One week after starting their first clinical attachment, all participants will be asked to complete a further short self-administered questionnaire (Questionnaire B), covering beliefs and plans regarding dermatitis prevention behaviours. At intervention-plus sites, it will also ask about use of, and views on, the BCP. Questionnaire B will be sent by post to participants with a business reply envelope for return to the SCIN research team.

To account for possible seasonal variation in the prevalence of dermatitis, final data collection will be carried out 12 months after baseline. All participants will be asked to answer a third self-administered questionnaire (Questionnaire C) and again to have their hands photographed. Questionnaire C will be distributed by the local field workers, and will cover: clinical attachments undertaken in the past year; hours worked per week over the past year; beliefs and plans regarding dermatitis prevention behaviours; participation in, and views about, the BCP (only at intervention-plus sites); activities outside work which predispose to hand dermatitis; recent practices regarding use of gloves; recent practices regarding hand cleansing; recent use of moisturising creams; history of hand dermatitis in the past 12 months (including its investigation and treatment, and any consequent loss of time from work or restriction of duties); and the EQ-5D questionnaire. Student nurses will be asked to place the completed Questionnaire C into a sealed business reply envelope for return to the SCIN team via the field worker or directly.

In addition, any attendance at OH with symptoms of hand/wrist dermatitis and any requests for extra provision of emollients during the study period will be documented.

#### Study group 2 – intensive care nurses

ICU nurses who agree to participate will be asked to complete a self-administered baseline questionnaire (Questionnaire A) similar to that for student nurses, but also including items on current occupation; recent practices regarding use of gloves; recent practices regarding hand cleansing; recent use of moisturising creams; and any sickness absence or modification of duties during the past 12 months because of hand dermatitis. Arrangements for the return of completed questionnaires will be as for student nurses. They will also have their hands and wrists photographed.

Once recruitment has been completed, field workers at intervention-plus sites will promote the importance of optimising equipment for hand cleansing, and dispensation of moisturising cream. The written leaflet about prevention of hand dermatitis will be made available to all staff on the ward (not only those individuals who have consented to take part in the study), and an email will be sent via the lead ICU nurses to all staff on the ward with a link to the BCP. Uptake of the BCP will be documented.

Following recruitment, all ICU nurses (not just those who have agreed to participate in the study) at intervention-light sites will also be given the written leaflet about prevention of hand dermatitis.

Two weeks after the BCP has been offered (or at a similar interval after recruitment in the intervention-light sites), participants will be asked to complete a second questionnaire (Questionnaire B) about beliefs regarding prevention of dermatitis and (at intervention-plus sites) participation in, and views on the BCP. Questionnaire B will be sent to participants by post with a business reply envelope. All participants at both the intervention-plus and intervention-light sites will be encouraged (orally, through the written advice leaflet and by email reminders sent out by the SCIN research team at 4 and 8 months) to attend the OH department at an early stage should they develop hand dermatitis. Email reminders will also contain a positive reinforcement message to encourage ongoing participation in the study. In addition, at the intervention sites, the email reminders will reinforce the BCP.

Outcomes will be assessed 12 months after baseline. All participants will be asked to complete a final self-administered questionnaire (Questionnaire C), and again to have their hands and wrists photographed. Questionnaire C will be sent out to field workers by the SCIN research team 2 months before the end of the study, and they in turn will pass them on to participants at the time that they recall them to have their hands photographed. The nurses will be asked to place the completed questionnaire into a sealed business reply envelope and return it via field worker or directly to the SCIN team in London.

### Withdrawal from the study

Participants who decide to withdraw from the study, for whatever reason, will be asked to complete a shortened version of Questionnaire C and will be invited to have follow-up photographs of their hands and wrists.

### Study instruments and data collection tools

#### Questionnaires

The study questionnaires have been developed specifically for this study, and piloted in a sample of 22 student nurses and 22 ICU nurses. Questions about dermatitis are derived from the Nordic Occupational Skin Questionnaire [37]. The EQ-5D health-related quality of life questionnaire [36] has been included and the questions on beliefs are based on the structure suggested for assessing TPB [38] and are informed by information we obtained from focus groups on student and ICU nurses.

Throughout the study, non-responders to any of the three study questionnaires will be sent an email reminder from the SCIN research team with a request that they complete and return the questionnaire directly to the SCIN team. If questionnaires remain outstanding, another copy of the paper questionnaire will be posted to the participant’s preferred postal address, along with a business reply envelope. If questionnaires remain outstanding after a further 2 weeks, participants will be sent up to two reminder text messages (or telephone messages if they have given a land line number).

At the end of the study period, participants who have completed and returned all three study questionnaires will be entered into a prize draw, giving them a chance to win one of the study cameras (a total of 26 cameras will be offered). Information about the draw and prizes will be included in the Participant Information Sheet.

#### Ascertainment of hand dermatitis and description of the photographic method

The protocol for photographing hands and wrists has been developed in consultation with a medical photographer and is consistent with the views required for the photographic assessment scale described by Coenraads [39]. It prescribes a method for obtaining repeatable standardised digital images that allow assessment of skin condition and colour.

As a last resort, where the local field worker is unable to make personal contact with a participant at follow-up, they will be offered an opportunity to send the SCIN research team ‘selfie’ photographs of their hands and wrists.

Initial assessment of photographs will be carried out by a dermatology research nurse (blind to intervention group), who will classify them as showing ‘definite/possible dermatitis’ or ‘no dermatitis’. All images classed as ‘definite/possible dermatitis’, together with a sample of 250 that are classed as ‘no dermatitis’ will then be assessed independently by two dermatologists. Final determination of whether or not dermatitis is present will be according to the opinion of the dermatologists, with any differences between them resolved by discussion. Provided the dermatologists do not identify a significant rate of dermatitis among the images that the nurse originally classed as ‘no dermatitis’, her assessment of ‘no dermatitis’ will be accepted for the images that were not selected for dermatologist assessment.

Our rationale for ascertaining any, and not only more severe hand dermatitis, is that even minimal hand dermatitis progresses to significant hand eczema in a cumulative way over time [39]. However, the dermatologists will also grade disease severity since severe hand dermatitis causes more distress and is associated with greater loss of time from work. We will use a method developed by Coenraads et al. [39] for classifying hand eczema severity: a simple categorization into five grades (clear, almost clear, mild, moderate, severe and very severe). Again, differences between the two dermatologists will be resolved by discussion.

### Data analysis

Outcomes will be assessed separately for the two study groups (student nurses and ICU nurses).

#### Outcome measures

For each study group, the principal outcome measure will be the difference between intervention-plus and intervention-light sites in the change in point prevalence of visible hand dermatitis from baseline to the end of follow-up.

Secondary outcomes will be the difference between intervention and control sites in:

- The difference between intervention and control sites in the change in prevalence and severity of visible hand dermatitis from baseline to the end of follow-up (as ascertained by the dermatologists)
- Days lost from sickness absence and days of modified duties because of hand dermatitis per 100 days of nurse time during the 12 months of follow-up
- The change from baseline to after completion of the BCP, and to the end of the 12-month follow-up in beliefs about dermatitis prevention behaviours
- The change from baseline to the end of follow-up in the reported frequency of: use of hand rubs for hand cleansing; hand-washing with water; and use of moisturising creams (for student nurses, who will not have started clinical attachments at the beginning of the study, this will reduce to differences between the intervention and control sites at the end of the follow-up)
- The change from baseline to the end of follow-up in quality of life score
- The use of moisturiser provided for the intervention (in terms of requests for further supplies by student nurses and orders for supplies of moisturisers by ICUs)

We will also document the reported participation in the BCP, reasons given for not participating, and comments on its content.

### Method of analysis

Differences in changes in the prevalence of the primary and secondary outcomes from baseline to follow-up between the two arms will be assessed by random-intercept multi-level models to account for possible clustering by site. For the primary outcome, the relative odds of having dermatitis at follow-up in the intervention-plus group as compared to the intervention-light group after adjusting for dermatitis at baseline will be estimated using a random-intercept logistic regression model. If inadvertent potentially confounding changes occur at some sites during the course of the study, we will carry out sensitivity analyses excluding the sites concerned. We will test whether differences between the intervention and control groups in dermatitis prevention behaviours are mediated by differences in beliefs and plans about the relevant behaviour.

### Economic analysis

In addition to analyses of clinical effectiveness, we will assess the cost-effectiveness of the interventions in the two staff groups from a healthcare and societal perspective. Healthcare costs will be calculated for the 12-month follow-up period and will be based on the number of contacts with clinical staff (occupational health, primary care staff, dermatologists, etc.) as a result of hand dermatitis, combined with appropriate unit cost information [40] and the cost of supplying moisturising creams (with costs obtained from the *British National Formulary* (*BNF*) for prescribed formulations). The service use information will be collected using a short self-report schedule based on the Client Service Receipt Inventory. These service costs will be added to the cost of the BCP itself, which will be based on development time and staff time accessing the package. Societal costs will be calculated by adding healthcare costs to the costs of lost production, based on days off work combined with wage rates. Cost comparisons between the groups will be made using bootstrapped regression models assuming that the cost data are skewed. Costs from both perspectives will be combined with the primary outcome measure in a cost-effectiveness analysis. If costs are reduced for one group and outcomes are better then it will be ‘dominant’. If one group has higher costs and better outcomes then incremental cost-effectiveness ratios (ICERs) will be generated, defined as the difference in costs divided by the difference in outcomes. Using the primary outcome measure, the ICER will indicate the extra cost incurred for one extra participant to be free of visible hand dermatitis in either hand at 12-month follow-up. There will be uncertainty around the cost and effectiveness estimates and this will be addressed using cost-effectiveness planes generated through repeated resampling from the data set to generate 1000 cost-outcome combinations and plotting these. To inform healthcare spending decisions it is helpful also to combine costs with quality-adjusted life years (QALYs). The most widely used QALY measure in England is the EQ-5D, which has been recommended for dermatological interventions [36]. This measure will be used at baseline and follow-up, and area under the curve methods used to produce QALYs. Similar analyses to those described above will be conducted to assess the relationship between costs and QALYs, but we will also generate cost-effectiveness acceptability curves (CEACs) to show the probability that the intervention is cost-effective for different threshold values placed on a QALY [41]. CEACs will still be produced but will be more exploratory; they will be used to identify threshold values where the likelihood of the intervention being cost-effective is 50 %, 60 %, 70 %, 80 % and 90 %). While standard unit costs are being used for most services it will be necessary to calculate the intervention costs specifically for the study. This will be based on estimates of staff time spent developing the intervention and staff time spent accessing it. We will increase/decrease both of these aspects in sensitivity analyses by 10–50 % to see the impact that these changes have on the probability that the intervention is cost-effective at the £20,000 and £30,000 QALY thresholds. Sensitivity analyses will also be conducted on the societal cost-effectiveness estimates by increasing/decreasing the cost of lost work time by 10–50 % and assessing the impact on the CEACs.

If the intervention is shown to be cost-effective then we will make estimates of the number of nurses who might access the intervention were it to become recommended practice. This will in turn allow us to estimate the budget impact for the NHS and the benefits in terms of total QALYs gained. Uncertainty around these estimates will be investigated through the sensitivity analyses describe above.

#### Process evaluation

We will collect data on and describe:

1. Uptake of the intervention, in terms of:
  Proportion of eligible nurses who access the online BCP
  Proportion of eligible nurses who complete the online BCP
2. Acceptability of the intervention in terms of:
  Perceived interest, relevance to role and likelihood of recommending it to colleagues

## Discussion

A range of operational and practical issues have arisen during the planning and implementation of the trial, including:

1. A large number of sites whose personnel had initially expressed an interest in taking part in the study were later unable to proceed due to various local issues (e.g. a subsequent reduction in OH staff to support the study or an increase in routine operational workload). As a consequence, it was necessary to invite additional OH services to take part in the study. This has enabled the research team to increase the total number of sites taking part in the study from the number initially planned
2. The recruitment rate in some sites has been lower than expected; therefore, to improve the number of participants taking part in the study, the research team have requested participating sites (where possible) to undertake a second round of recruitment. In addition, the research team agree to broaden the inclusion criteria for ‘critical care nurses’ to also include ‘special care baby unit’ nurses:
3. A dedicated full-time research nurse has been recruited to the central research. Their role will be to assist with further recruitment and capturing follow-up data at participating sites

### Trial status

At the time of submission, the main trial has been underway since September 2014, with final recruitment planned for March 2016. The trial will be completed no later than September 2017 (pending approval from the funder).

## Abbreviations

BCP: behavioural change programme
CEACs: cost-effectiveness acceptability curves
CONSORT: Consolidated Standards of Reporting Trials
CTU: Clinical Trials Unit
EQ-5D: EuroQol-5D
HSE: Health and Safety Executive
HTA: Health Technology Assessment
ICERs: incremental cost-effectiveness ratios
ICU: intensive care units
MRC: Medical Research Council
MRSA: methicillin-resistant *Staphylococcus aureus*
NHS: National Health Service
NICE: National Institute for Health and Clinical Excellence
NIHR: National Institute for Health Research
OH: occupational health
QALYs: quality-adjusted life years
RCN: Royal College of Nursing
RCT: randomised controlled trial
TEWL: transepidermal water loss
TPB: Theory of Planned Behaviour
UKCRC: UK Clinical Research Collaboration
WHO: World Health Organization
